# Supplementary material for: Deciphering the state of immune silence in fatal COVID-19 patients
Source: Nat Commun. 2021 Mar 5;12:1428. doi: 10.1038/s41467-021-21702-6 (PMC7935849; doi:10.1038/s41467-021-21702-6)
Supplement: Supplementary file 1 — Supplementary Information [file 41467_2021_21702_MOESM1_ESM.pdf]

SUPPLEMENTARY INFORMATION.
Deciphering the state of immune silence in fatal COVID-19 patients

Bost P., et al

Supplementary Figure 1, Bost et al.

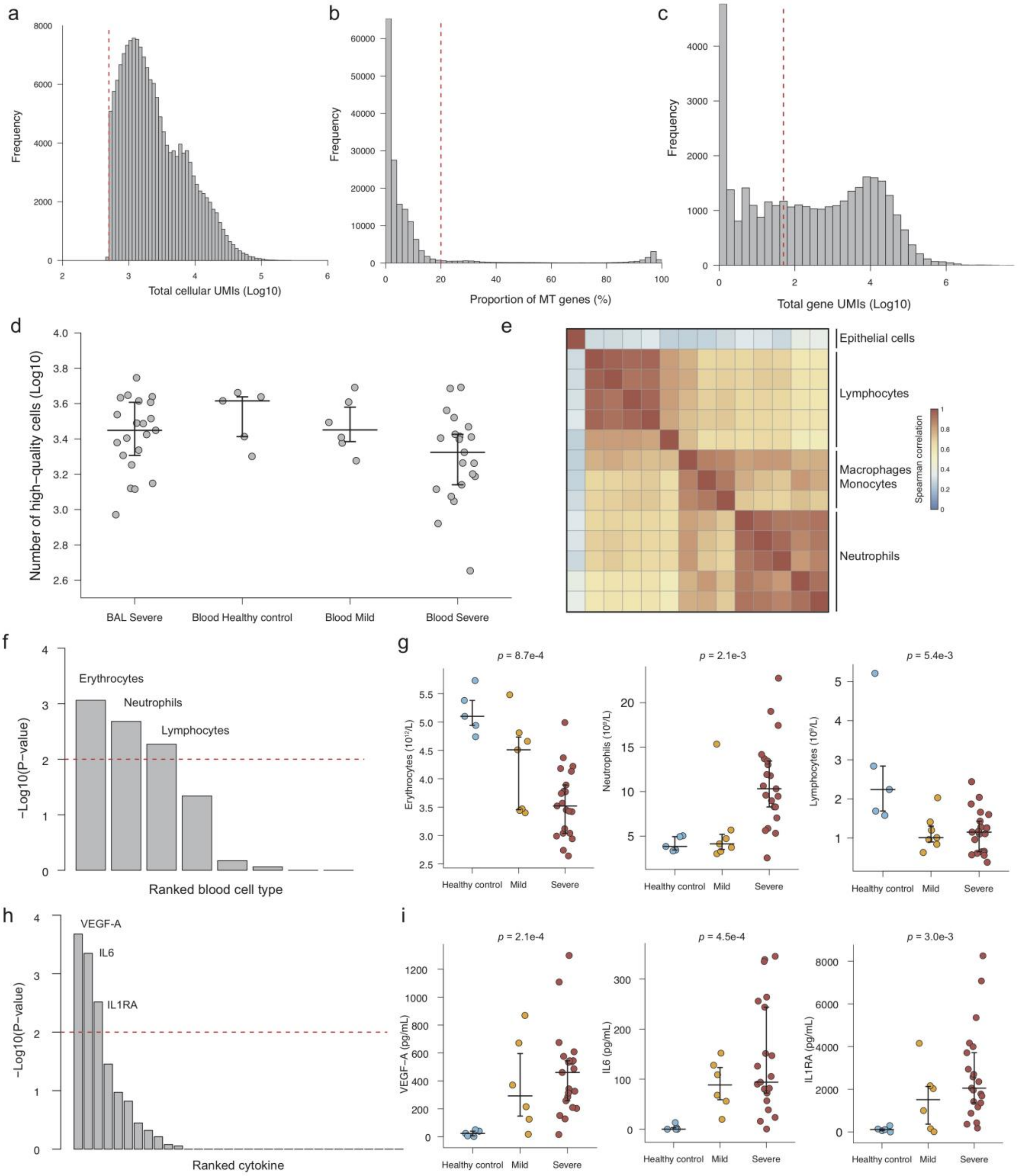

Supplementary Figure 1. Quality control of the scRNA-seq dataset and blood cell count analysis. (a) Distribution of total cellular unique molecular identifier (UMIs, log10 scale). 500 UMI threshold (vertical red dashed line) filters out low quality cells. **(b)** Proportion of mitochondrial UMIs among total cellular UMIs. 20% threshold (vertical red dashed line) filters out low quality cells. **(c)** Distribution of total gene UMIs (log10 scale). 50 UMI threshold (vertical red dashed line) filters out lowly expressed genes. N= 147207 cells from 32 independent patients were examined (a-c). **(d)** Number of high quality cells that passed QC in the different samples types. Median and 5%-95% theoretical quantiles are shown. N= 53 independent clinical samples were used (21 matched BAL and blood samples from severe patients, 5 and 6 blood samples from healthy controls and mild patients, respectively). **(e)** Spearman's correlation heatmap of the mean expression of the most variable genes in each single-cell cluster. **(f)** Effects of patient clinical status on blood cell counts. The p-values are computed by fitting and testing an ANOVA model (Methods, one-sided Fisher test). 0.01 significance threshold (vertical red dashed line) identifies cell types which blood count is affected by the clinical status. **(g)** Number of erythrocytes (left panel), neutrophils (middle panel) and lymphocytes (right panel) in blood based on patients clinical status. Displayed *p*-values were computed as described above, by fitting and testing an ANOVA model (one-sided Fisher test). Median and 5%-95% theoretical quantiles are shown. N= 33 independent clinical samples (5 healthy controls, 7 mild, 21 severe patients). **(h)** Effects of patient clinical status on serum cytokine concentration. The *p*-values are computed by ANOVA function (Methods, one-sided Fisher test). The vertical red dashed line corresponds to the 0.01 significance threshold used to identify cytokines which serum concentration is affected by the clinical status. **(i)** Serum concentration of VEGF-A (left panel), IL6 (middle panel) and IL1RA (right panel) based on patients clinical status. Displayed p-values were computed as described above, by ANOVA function (one-sided Fisher test). Median and 5%-95% theoretical quantiles are shown. (f, h,i) N= 32 independent clinical samples (5 healthy controls, 6 mild, 21 severe patients).

Supplementary Figure 2, Bost et al.

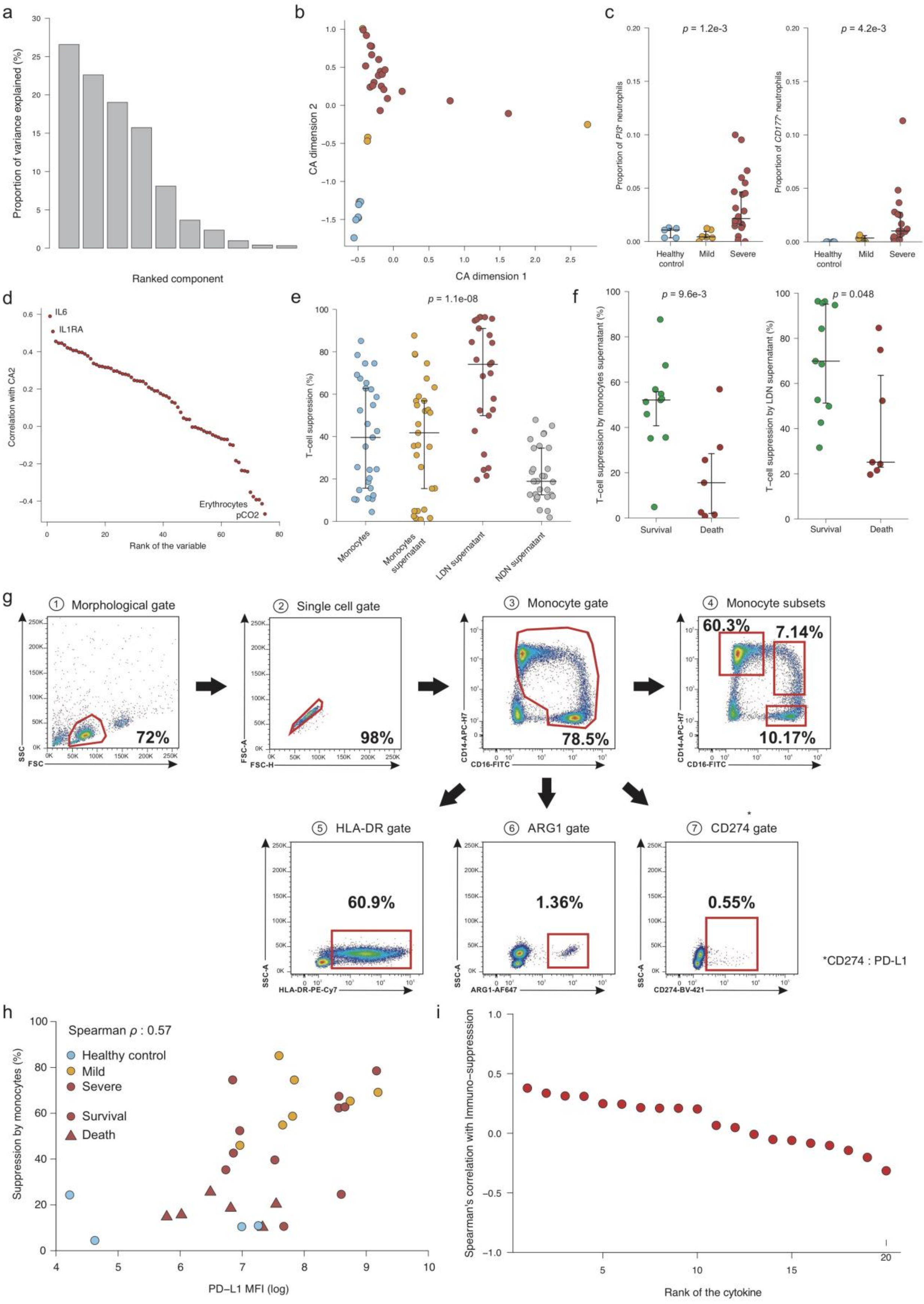

Supplementary Figure 2. Dissection of the blood neutrophil compartment. (a) Percentage of variance explained by the 10 first components of the blood neutrophil CA. N= 32 independent clinical samples were used, including 5 derived from healthy patients, 6 from mild patients and 21 from severe patients. **(b)** Correspondence Analysis of blood neutrophils (first and second components). **(c)** Proportion of *PI3*$^+$ (left panel) and *CD177*$^+$ neutrophils (right panel) among total blood neutrophils based on patient clinical status. A one-sided Kruskall-Wallis rank test was used in both panels. Median and 5%-95% theoretical quantiles are shown. N= 32 independent clinical samples were used, including 5 derived from healthy patients, 6 from mild patients and 21 from severe patients. **(d)** Pearson correlation of biological variables with blood neutrophil CA dimension 2. **(e)** T-cell suppression by various blood myeloid cell or their supernatant. The p-value is computed by fitting and testing an ANOVA model (one-sided Fisher test, $F = 16.0$, degree of freedom equal to 3). Median and 5%-95% theoretical quantiles are shown. N=112 independent measurements were used. **(f)** T-cell suppression by monocyte supernatant (left panel) and LDN supernatant (right panel) of severe patients based on clinical outcome. *P*-values are computed by applying a two-sided Welch's t-test (t=3.0 and t=2.2 with a degree of freedom of 12.9 and 11.6 respectively). Median and 5%-95% theoretical quantiles are shown. N=18 independent clinical samples were used, including 11 derived from patients who survived and 7 from deceased patients (**g**) Flow cytometry gating used to measure HLA-DR, ARG1 and PD-L1 (CD274) expression in monocytes. **(h)** Association between monocytes PD-L1 MFI and suppression. **(i)** Pearson correlation of monocyte secreted cytokine concentrations with monocyte immune-suppression.

Supplementary Figure 3, Bost et al.

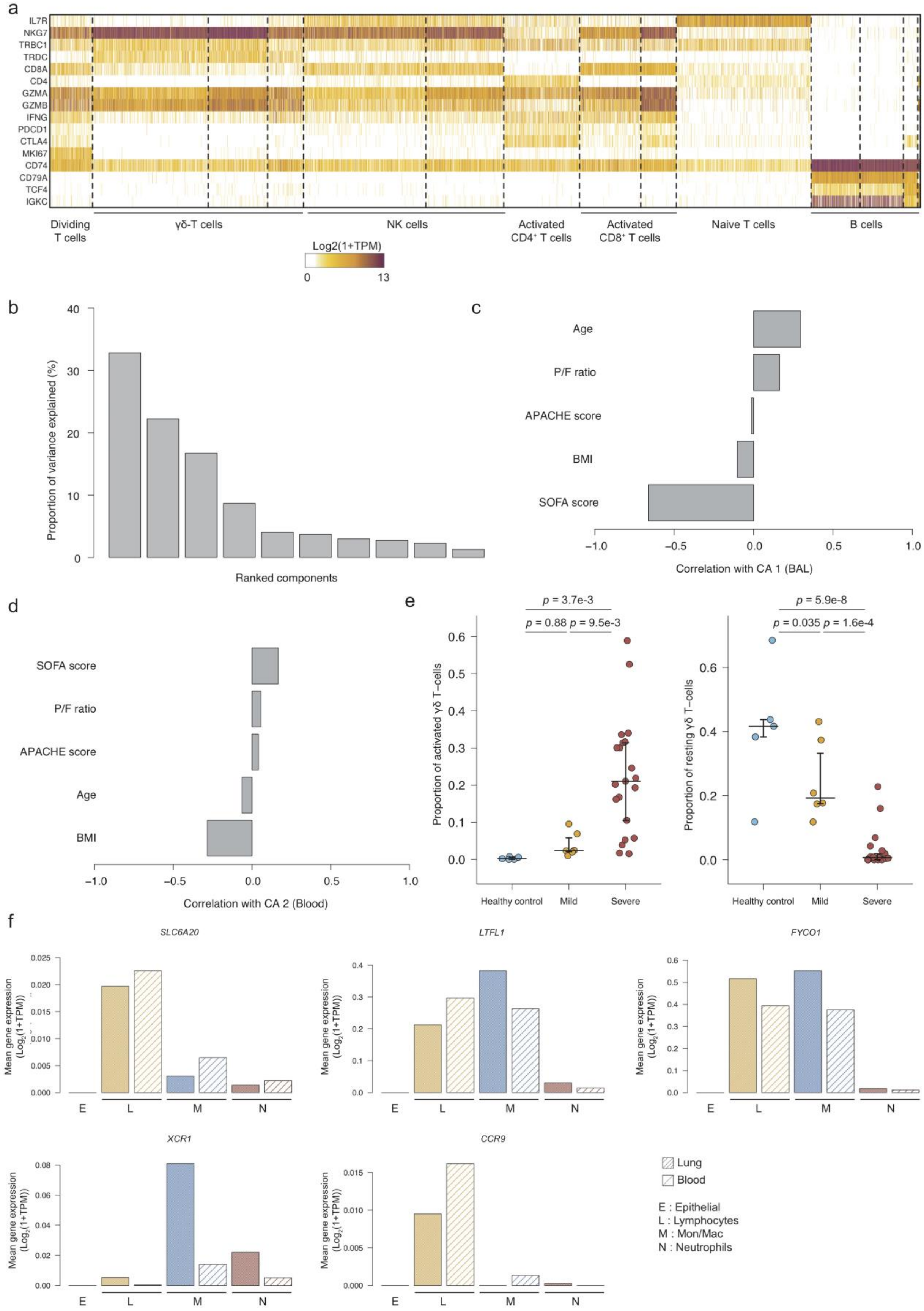

Supplementary Figure 3. Association between blood and BAL lymphocyte populations and patient clinical status. (a) Expression heatmap of lymphocytes. The 16 displayed genes correspond to genes known to play an important role in lymphocyte biology. **(b)** Percentage of variance explained by the 10 first components of the lymphocyte CA. N= 32 independent clinical samples were used, including 5 derived from healthy patients, 6 from mild patients and 21 from severe patients. **(c)** Correlation between CA dimension 1 score of BAL samples and 5 clinical variables. **(d)** Correlation between CA dimension 2 score of blood samples and 5 clinical variables. **(e)** Proportion of resting (left) and activated (right) γδ-T cells among blood lymphocytes. A one-sided Tukey's range test was used to compute p-values. Median and 5%-95% theoretical quantiles are shown. N= 32 independent clinical samples were used, including 5 derived from healthy patients, 6 from mild patients and 21 from severe patients. **(f)** Expression of five severe COVID-19 genetically associated genes among tissues and cell types. N=61989 cells derived from 21 independent patients were used.

Supplementary Figure 4, Bost et al.

a

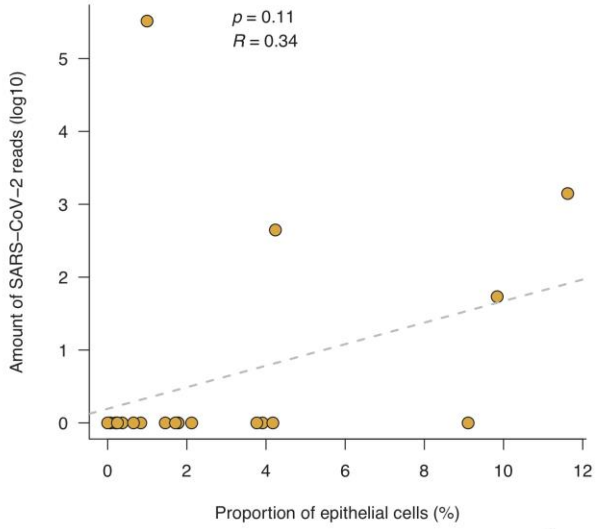

b

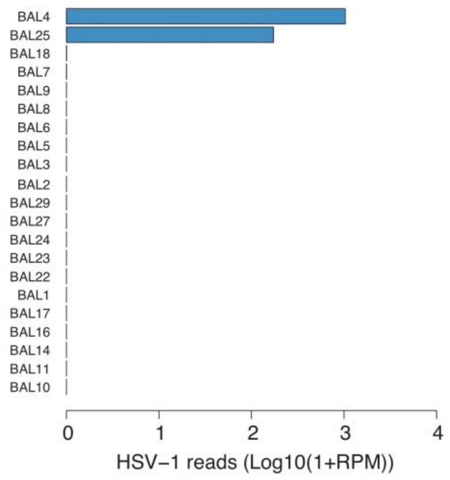

c

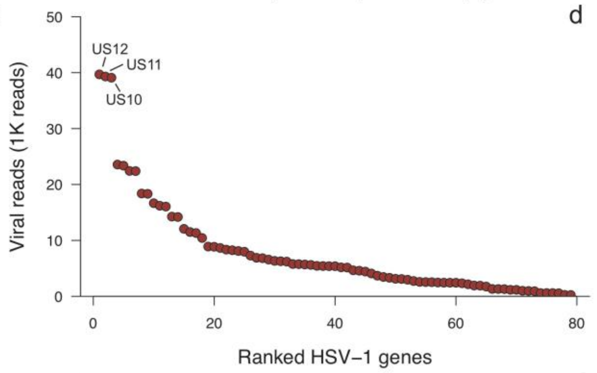

d

Patient	HSV-1	HSV-2	HCMV	VZV
**8**	Undetected	Undetected	Undetected	Undetected
**13**	Undetected	Undetected	Undetected	Undetected
**25 (Time 1)**	Ct = 17.4	Undetected	Undetected	Undetected
**25 (Time 2)**	Ct = 12.67	Undetected	Undetected	Undetected

e

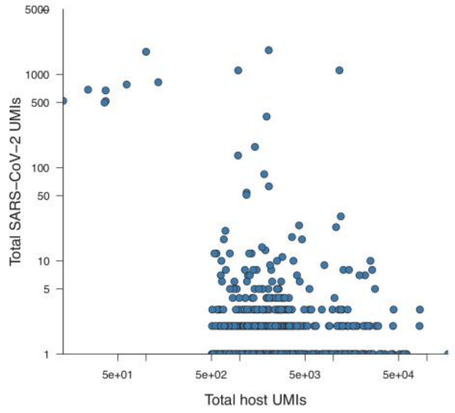

f

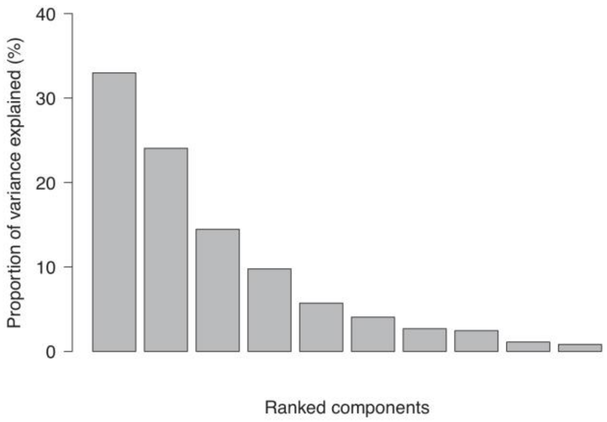

g

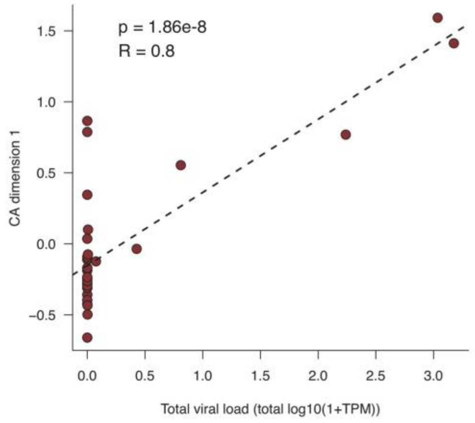

Supplementary Figure 4. Study of the pulmonary viral landscape in severe COVID-19 patients. **(a)** Association between the proportion of epithelial cells in each BAL sample and the number of SARS-CoV-2 reads. The dashed line corresponds to a linear regression which *p*-value and *R* are displayed on the top of the panel. **(b)** Quantification of HSV-1 reads across BAL samples. **(c)** Ranked mean expression of HSV-1 genes in patients 4 and 25. **(d)** Results of the qPCR test for herpes viral infections. **(e)** Relationship between total host and SARS-CoV-2 UMIs in patient 8 BAL cells. **(f)** Percentage of variance explained by the 10 first components of the total blood CA. **(g)** Association between total viral load in the BAL and total blood CA dimension 1. The dashed line corresponds to a standard linear regression and its associated p-value and R are displayed in the panel.

Supplementary Table 1: Absolute cell numbers of immune cells identified in each patient's specimen referred to Figure 1C.

Healthy Controls	Epithelial cells	γδ-T cells	NK cells	CD4+ T cells	CD8+ T cells	B cells	Monocytes	Macrophages (1)	Macrophages (2)	Neutrophils (1)	Neutrophils (2)	Neutrophils (3)	Neutrophils (4)	Neutrophils (5)
Blood_38	2	219	696	191	1	426	230	11	0	21	564	2	10	29
Blood_39	2	357	194	88	0	138	227	20	0	37	1357	8	43	34
Blood_40	1	474	491	89	4	125	344	19	0	62	1067	3	27	53
Blood_41	1	176	138	24	0	60	109	3	0	12	402	28	11	18
Blood_42	1	447	69	35	6	63	204	15	0	25	605	15	4	6
Mild Patients	Epithelial cells	γδ-T cells	NK cells	CD4+ T cells	CD8+ T cells	B cells	Monocytes	Macrophages (1)	Macrophages (2)	Neutrophils (1)	Neutrophils (2)	Neutrophils (3)	Neutrophils (4)	Neutrophils (5)
Blood_32	0	51	31	47	1	2	418	4	0	85	1472	95	47	0
Blood_33	0	133	173	49	4	117	275	1	0	7	217	2653	18	11
Blood_34	1	175	121	307	13	86	232	10	1	23	268	36	3	0
Blood_35	1	150	55	78	7	42	333	4	0	37	1285	132	47	20
Blood_36	0	249	525	72	6	36	1058	5	0	301	13	4	3	1
Blood_37	0	150	208	85	8	91	241	5	0	54	140	5	8	5
Severe Patients	Epithelial cells	γδ-T cells	NK cells	CD4+ T cells	CD8+ T cells	B cells	Monocytes	Macrophages (1)	Macrophages (2)	Neutrophils (1)	Neutrophils (2)	Neutrophils (3)	Neutrophils (4)	Neutrophils (5)
BAL_01	11	0	0	1	0	0	12	273	0	8	218	62	434	293
BAL_02	182	131	135	255	796	2	0	2139	5	95	14	4	178	4
BAL_03	5	3	0	1	6	0	1	186	2	37	72	20	1853	184
BAL_04	72	6	18	20	63	3	0	168	0	144	3	2	35	27
BAL_05	5	0	6	1	22	0	2	406	2	83	73	3	1128	249
BAL_06	92	12	3	56	33	0	3	200	1	41	28	7	365	21
BAL_07	79	1	1	8	10	2	11	907	0	35	12	39	2053	1260
BAL_08	14	3	0	6	51	0	5	97	0	807	37	2	347	13
BAL_09	59	9	5	74	177	1	14	765	104	333	324	25	1382	107
BAL_10	7	15	0	40	164	1	10	215	1541	50	123	7	768	95
BAL_11	19	2	6	2	16	0	2	345	1	75	39	1	598	180
BAL_14	208	5	18	22	55	5	10	449	2	220	54	3	558	94
BAL_16	2	6	0	11	10	2	4	583	3	31	35	0	1669	440
BAL_17	10	2	3	17	9	1	4	742	6	39	349	52	2907	141
BAL_18	12	0	0	0	3	0	8	133	0	14	108	32	174	2783
BAL_22	161	12	17	101	171	4	1	173	76	910	180	8	2237	18
BAL_23	210	63	48	1201	1085	38	2	161	2012	48	38	2	185	262
BAL_24	111	1	0	4	27	0	14	389	15	607	128	15	1182	101
BAL_25	46	22	14	34	29	6	16	298	10	256	40	6	760	8
BAL_27	218	14	10	50	43	1	5	196	5	115	101	13	614	44
BAL_29	20	7	3	6	10	0	2	220	6	435	2	1	1601	84
Blood_01	0	32	44	18	1	130	190	4	0	9	1185	242	10	80
Blood_02	0	58	24	27	6	55	270	0	0	154	649	186	3	0
Blood_03	0	6	18	9	0	41	139	2	0	65	1138	412	13	9
Blood_04	0	179	124	36	61	70	347	2	0	1782	251	53	1	8
Blood_05	0	32	33	15	9	26	161	1	0	120	420	51	5	3
Blood_06	0	5	9	37	0	41	80	0	0	17	116	21	2	2
Blood_07	0	52	99	10	0	5	122	1	1	5	554	267	15	1
Blood_08	2	144	7	78	30	1	159	0	0	2009	613	341	3	1
Blood_09	0	52	16	108	0	29	253	3	0	53	321	62	5	0
Blood_10	1	141	36	143	0	154	462	5	0	100	2145	114	4	3
Blood_11	0	12	44	48	1	12	257	5	0	476	297	116	8	11
Blood_14	0	41	62	58	2	17	188	4	0	126	43	8	0	1
Blood_16	2	398	134	29	4	99	331	3	0	15	717	221	8	1
Blood_17	0	1	38	6	0	25	260	0	0	96	1356	230	1	1
Blood_18	0	37	107	27	1	42	325	2	0	6	1411	908	2	5
Blood_22	0	21	28	35	0	20	136	4	0	16	936	60	2	0
Blood_23	0	56	70	84	0	51	515	7	0	20	1083	123	29	30
Blood_24	0	34	24	12	2	24	243	1	0	610	463	102	2	0
Blood_25	0	83	82	21	1	40	206	0	0	300	128	37	0	0
Blood_27	4	26	78	24	0	26	191	0	0	98	1739	223	5	0
Blood_29	0	25	19	8	0	5	112	1	0	131	206	741	8	4
