# Supplementary material for: Deciphering the state of immune silence in fatal COVID-19 patients
Source: Nat Commun. 2021 Mar 5;12:1428. doi: 10.1038/s41467-021-21702-6 (PMC7935849; doi:10.1038/s41467-021-21702-6)
Supplement: Supplementary file 3 — Descriptions of Additional Supplementary Files [file 41467_2021_21702_MOESM3_ESM.pdf]

Descriptions of Additional Supplementary Files

Supplementary data 1

Description: Gene expression profile of the main 5 neutrophil clusters identified in the study.
