# Supplementary material for: Deciphering the state of immune silence in fatal COVID-19 patients
Source: Nat Commun. 2021 Mar 5;12:1428. doi: 10.1038/s41467-021-21702-6 (PMC7935849; doi:10.1038/s41467-021-21702-6)
Supplement: Supplementary file 5 — Reporting Summary [file 41467_2021_21702_MOESM5_ESM.pdf]

nature research

Corresponding author(s): Ido Amit
Vincenzo Bronte

Last updated by author(s): 01/14/2021

Reporting Summary

Nature Research wishes to improve the reproducibility of the work that we publish. This form provides structure for consistency and transparency in reporting. For further information on Nature Research policies, see our Editorial Policies and the Editorial Policy Checklist.

Statistics

For all statistical analyses, confirm that the following items are present in the figure legend, table legend, main text, or Methods section.

n/a	Confirmed	
☐	☒	The exact sample size (n) for each experimental group/condition, given as a discrete number and unit of measurement
☐	☒	A statement on whether measurements were taken from distinct samples or whether the same sample was measured repeatedly
☐	☒	The statistical test(s) used AND whether they are one- or two-sided<br>*Only common tests should be described solely by name; describe more complex techniques in the Methods section.*
☐	☒	A description of all covariates tested
☐	☒	A description of any assumptions or corrections, such as tests of normality and adjustment for multiple comparisons
☐	☒	A full description of the statistical parameters including central tendency (e.g. means) or other basic estimates (e.g. regression coefficient) AND variation (e.g. standard deviation) or associated estimates of uncertainty (e.g. confidence intervals)
☐	☒	For null hypothesis testing, the test statistic (e.g. F, t, r) with confidence intervals, effect sizes, degrees of freedom and P value noted<br>*Give P values as exact values whenever suitable.*
☒	☐	For Bayesian analysis, information on the choice of priors and Markov chain Monte Carlo settings
☒	☐	For hierarchical and complex designs, identification of the appropriate level for tests and full reporting of outcomes
☐	☒	Estimates of effect sizes (e.g. Cohen's d, Pearson's r), indicating how they were calculated

Our web collection on statistics for biologists contains articles on many of the points above.

Software and code

Policy information about availability of computer code

Data collection	ScRNA-seq expression data were deposited in the Gene Expression Omnibus (GEO) under accession code GSE157344. In this work we did not use any code generated by work other than our. We generated the code from our study which is available at https://github.com/PierreBSC/Verona_COVID19 .
Data analysis	Generation of UMI tables. Upstream processing of reads was done using the CellRanger toolkit with default parameters. SARS-CoV-2 (NCBI reference number: NC_045512.2) and human hg38 ge-nomes were downloaded from NCBI website. SARS-CoV-2 GTF annotation file was downloaded from the UCSC and merged with the human GTF as an additional chromosome. ORF_10 Gene 3' boundary extended by 100 bases to catch all reads that belong to this transcript.<br><br>ScRNA-seq expression data were deposited in the Gene Expression Omnibus (GEO) under accession code GSE157344.<br>We generated the code from our study which is available at https://github.com/PierreBSC/Verona_COVID19 .<br><br>High-level analysis of scRNA-seq expression data. ScRNA-seq expression data analysis were per-formed using the R-based Pagoda2 pipeline (https://github.com/hms-dbmi/pagoda2/)25 in addition to an in-house R script. Briefly UMI table were loaded using the read.10x.matrices() function. Low quality cells were removed using the following strategy: cell with less than 500 UMIs and more than 20% of mitochondrial genes were removed. Two rounds of analysis were performed: in the first one, all filtered cells were used to identify the major cell types, then cells from each cellular compartments are analyzed individually to provide more detailed informations. For each analysis, the number of Highly Variables Genes (HVGs) was determined using the adjustVariance() function with the gam parameter set to 10. HVGs were selected using the following strategy: for each gene, its number of zeros and its mean expression are computed. A local polynomial model is then used to predict the number of zeros according to the log mean expression (loess function with degree pa-rameter set to 2). The residuals of this model (excess of zeros) are then used to ranked the genes and the genes with the highest excess of the zeros are considered as the most HVGs. PCA reduction is then computed using the calculatePcaReduction() function. The number of computed PC was changed in each analysis due to variable number of cells and cellular heterogeneity. A K-nearest neighbor graph was then build with the function makeKnnGraph() with

the K value set to 30 and the distance parameter set to ‘cosine’. In order to get high-quality cell clusters, we used the Leiden community detection implemented in the R package leiden, a wrapper of the python package leidenalg. The leiden() function was applied to the KNN graphs with default parameters for each analysis. Marker genes were identified using the getDifferentialGenes() function. UMAP low di-mensional embedding was computed using the uwot R package, and more precisely the umap() function with the n_neighbors parameter set to 30, and the metric parameter set to ‘cosine’. In order to group clusters of cells in the first round of analysis, mean gene expression of the most variable genes was computed using the aggregate() function. Spearman’s correlation matrix was then com-puted using the cor() function with the method parameter set to ‘Spearman’. Hierarchical clustering was then performed on this matrix using Ward’s method and the resulting tree used to aggregate the cell clusters.

Correspondence Analysis of the scRNA-seq data. In order to identify trends in cellular composition across samples we used a multivariate technique called Correspondance Analysis (CA). CA is highly similar to PCA but is applied to contingency table instead of classical continuous data table. First data are pre-processed by dividing each entry by the sum of all matrix entries resulting in the matrix S. Then a second matrix is computed by subtracting the expected distribution of samples (obtained by multiplying the row and column marginal probably vectors) resulting in a new matrix M. M is then decomposed using singular value decomposition. Because CA is a descriptive technique, it has the advantage of being applicable to tables whether or not the chi-squared statistic is appropriate. We used the R implementation of CA from the package FactoMineR (CA() function) with default parameters. To determine the significant components we looked at the scree plot and selected the eigenvalues/component located before the elbow. To improve the quality of our analysis, we re-moved cell clusters corresponding to red blood cells, platelets and cancer cells from patient 8.

To detect clinical and biological variables associated with the computed correspondance compo-nents we used the following strategy: for cytokine concentrations, we first took the square root of the initial values to get normally distributed variables and then computed Pearson’s correlation with each component independently. For the other continuous variables (clinical scores, age, BMI…), Pearson’s correlation was directly computed. To test the association between CA component or a specific cell type proportion and a categorical variable (i.e. patient clinical status and survival) we either applied a Tukey's range test (TukeyHSD() function) if the variable is not heavy tailed. If the cell proportions are clearly heavy-tailed, we applied a Kruskall-Wallis rank test. Normality of the variables was checked using the Shapiro-Wilk test, through the R function shapiro.test().

Viral-Track analysis. To detect and study viruses in our scRNA-seq samples we used Viral-Track, a computational tool that screen the raw sequencing files to find viral reads (32479746). As previous-ly described, processing of the file was performed using UMI-tool (28100584). First, cell barcodes were extracted and a putative whitelist computed using the umi_tools whitelist command with the parameters ‘–stdin —bc-pattern = CCCCCCCCCCCCCCCCNNNNNNNNNN –log2stderr’. Fol-lowing the mapping of the reads to viral genomes and transcript assembly, the mapped reads were assigned to transcripts using the R package Rsubread through the function featureCounts() with de-fault parameters. The command ‘umi_tools count’ is then used to compute the final expression table with the following parameters:–per-gene–gene-tag = XT–assigned-status-tag = XS–per-cell.
In the case of patient 8, cells were not filtered on total host UMIs and proportion of MT UMIs but only on total combined host and viral UMIs to avoid removing apoptotic cells containing a high vi-ral load but expressing few host genes.

Quantification of HSV-1 gene expression. Transcriptome annotation file for the NC_001806 viral segment was manually downloaded from the NCBI server. BAM files containing the HSV-1 reads from patients 4 and 25 were loaded into R using the GenomicAlignments package and gene expres-sion quantification done using the featureCounts() function from the Rsubread package with de-fault parameters.
Analysis of the serum cytokine, blood cell count and clinical data. Using a Cullen and Frey graph (descdist() function from the fitdistrplus package)we observed that both serum cytokine and blood cell count variables could be transformed into gaussian-like variables by applying a simple square root function and then used for further analysis. Association between blood cell counts or serum cytokine concentration and patient clinical status was assessed by fitting an ANOVA model to the transformed variables (aov() and anova() functions). Correction for multiple testing was done using the p.adjust function with parameter method set to ‘BH’. When correlations with a CA dimension were computed, the cor() function with default parameters was used. To validate the association between the SOFA score and the lymphoid CA dimension 1 we fitted a basic linear model with the lm() function and assessed the significance of the association by performing a Fisher test with the anova() function.

Analysis of the immuno-suppression, flow cytometry and cytokine secretion data. As both both flow cytometry and cytokine secretion data were extremely heavy-tailed we applied a logarithmic trans-formation with a pseudo count of 1 (log10(1+x)). Spearman correlations between protein MFI or cytokine concentration and immune-suppression was computed using the cor() function.
In order to model the relationship between ARG1 MFI and immune-suppression we applied a func-tion similar to the Hill function used in biochemistry and to model drug dose-response curves.

Here S corresponds to the immune-suppression, x to the transformed ARG1 MFI, Emin to the basal immune-suppression, Emax to the maximal suppression that can be induced by ARG1, K to the trans-formed ARG1 MFI required to get half of the maximal suppression (Emin +Emax) and n the coopera-tion coefficient. This function was fitted using the nls() function with default parameters.

Quantitative variables indicated in table 1 were expressed as the median and interquartile range (IQR), qualitative ones as percentages. All statistical analyses were performed using R 3.6.1 on an Ubuntu 18.04 workstation.

FlowJo software v.10 (Tree star, Inc, Ashland,OR, USA) was used for flow cytometry data analysis

For manuscripts utilizing custom algorithms or software that are central to the research but not yet described in published literature, software must be made available to editors and reviewers. We strongly encourage code deposition in a community repository (e.g. GitHub). See the Nature Research guidelines for submitting code & software for further information.

Data

Policy information about availability of data

All manuscripts must include a data availability statement. This statement should provide the following information, where applicable:

- Accession codes, unique identifiers, or web links for publicly available datasets
- A list of figures that have associated raw data
- A description of any restrictions on data availability

The authors declare that all the other data supporting the findings of this study are available within the article and its supplementary information files and from the corresponding authors upon request. ScRNA-seq expression data were deposited in the Gene Expression Omnibus (GEO) under accession code GSE157344. Figures associated with available raw data are: Fig 2e, S2e, S2f; Fig.2f, 2g s2g; Fig s2i; Fig 3e;Fig4e.

Field-specific reporting

Please select the one below that is the best fit for your research. If you are not sure, read the appropriate sections before making your selection.

☒ Life sciences ☐ Behavioural & social sciences ☐ Ecological, evolutionary & environmental sciences

For a reference copy of the document with all sections, see nature.com/documents/nr-reporting-summary-flat.pdf

Life sciences study design

All studies must disclose on these points even when the disclosure is negative.

Sample size	The study was designed as an observational trial. This study includes a group of 21 severe COVID-19 patients admitted to ICU, 10 mild SARS-CoV-2 patients and 5 HDs. The clinical features of the 3 groups of individuals are recapitulated in table 1. All 31 patients with COVID-19 were admitted, within the period from March 12th to April 20th 2020 to the University Hospital of Verona. At sampling, the stage of disease was categorized as mild (patients not requiring non- invasive/mechanical ventilation and/or admission to ICU, WHO ordinal score 3-4) or severe (patients requiring admission to ICU and/or non-invasive/mechanical ventilation, WHO ordinal score 6-7). All patients were hospitalized in ICU for respiratory organ failure as proved by their clinical parameters (SOFA score, pCO2, pO2, FiO2, P/F ratio). The sample size was not predetermined. We analyzed all the available samples in that time window.
Data exclusions	Patients that withdraw informed consent and indiviaduals younger than 18 years old were excluded from the study.
Replication	Not applicable to our clinical study, set to collect biological fluids at the time of positive Sars-Cov-2 infection only.
Randomization	Randomization was not applied to this study. Considering the catastrophic health and social crisis that COVID-19 generated in the Northern part of Italy at the time of the study began (March 12th to April 20th 2020), any patient admitted to the hospital, proved to be positive for Sars-Cov-2 and older that 18 years of age, was elegible to be enrolled in our study, providing the signed informed consent.
Blinding	The medical staff and researchers that collected the samples were awared of the severity of the disease (severe, mild and healthy donors). However the data collection and analysis were blinded.

Reporting for specific materials, systems and methods

We require information from authors about some types of materials, experimental systems and methods used in many studies. Here, indicate whether each material, system or method listed is relevant to your study. If you are not sure if a list item applies to your research, read the appropriate section before selecting a response.

Materials & experimental systems

n/a	Involved in the study	
☐	☒	Antibodies
☐	☒	Eukaryotic cell lines
☒	☐	Palaeontology and archaeology
☒	☐	Animals and other organisms
☐	☒	Human research participants
☐	☒	Clinical data
☒	☐	Dual use research of concern

Methods

n/a	Involved in the study	
☒	☐	ChIP-seq
☐	☒	Flow cytometry
☒	☐	MRI-based neuroimaging

Antibodies

Antibodies used	APC-H7-conjugated CD14 (clone Mphi9) cat. num. 560180, BD Biosciences, 5 µl test; PE-conjugated CD56 (clone NCAM16.2), 25 µl/ml, cat. num. 335791 BD Bioscinces; FITC-conjugated-CD16 (clone 3G8), cat. num. 555406 BD Biosciences, 20 µl test; PerCP-Cy5.5-conjugated CD3 (clone UCHT1), cat. num. 560835, BD Biosciences, 5 µl test; PE.Cy7-conjugated HLA-DR (clone L243), BD Biosciences

335795, 5 µl test; Brilliant Violet 421™-conjugated PD-L1 (clone MIH1), BD Biosciences 563738, 5 µl test; HRPconjugated anti-human-IgG, Thermo Scientific 31410, 0.8 mg/ml, 1:1000; HRP-conjugated anti-human-IgA, Thermo Scientific SA1-35467, 1.2 mg/ml, 1:1000; CD66b-FITC antibody, BD Biosciences 561927, 20 µl test; Purified anti-CD3 (clone OKT-3); Purified anti-CD28 (clone 28.2), Thermo Scientific 16-0289-81, 1 µg/test, 1 mg/ml; anti-CD3-PE/Cy7 (clone UCHT1), BD biosciences 563423, 5 µl/test; Purified Anti-ARG1 (clone 1.10); 1 mg/ml, 1:1000; Purified Mouse IgG1 κ Isotype Control, Clone MOPC-21, 1 mg/ml, 1:1000.

Validation

Home made Purified Anti-ARG1 (clone 1.10) IgG1 was validated in Trovato, R., et al., "Immunosuppression by monocytic myeloidderived suppressor cells in patients with pancreatic ductal carcinoma is orchestrated by STAT3." J Immunother Cancer, 2019. 7(1): p.255.

For 560180, BD Biosciences: Bernstein ID, Self S. Joint report of the Myeloid Section of the Second International Workshop on Human Leukocyte Differentiation Antigens. In: Reinherz EL, Haynes BF, Nadler LM, Bernstein ID, ed. Leukocyte Typing II: Human Myeloid and Hematopoietic Cells. New York, NY: Springer-Verlag; 1986:1-25. (Biology)

For 335791 we did not find any information from the company.

For 555406, Wirthmueller U, Kurosaki T, Murakami MS, Ravetch JV. Signal transduction by Fc gamma RIII (CD16) is mediated through the gamma chain. J Exp Med. 1992; 175(5):1381-1390.

For 560835, Beverley PC, Callard RE. Distinctive functional characteristics of human "T" lymphocytes defined by E rosetting or a monoclonal anti-T cell antibody. Eur J Immunol. 1981; 11(4):329-334.

For 335795, Grouard G, Durand I, Filgueira L, Banchereau J, Liu YL. Dendritic cells capable of stimulating T cells in germinal centres. Nature. 1996;384:364-367.

For 563738, Freeman GJ, Long AJ, Iwai Y, et al. Engagement of PD-1 immunoinhibitory receptor by a novel B7 family member leads to negative regulation of lymphocyte activation. J Exp Med. 2000; 192:1027-1034.

For 31410, A cellular model reflecting the phenotypic heterogeneity of mutant HRAS driven squamous cell carcinoma. Cantariño N,Fernández-Figueras MT,Valero V,Musulén E,Malinverni R,Granada I,Goldie SJ,Martín-Caballero J,Douet J,Forcales SV,Buschbeck M. International journal of cancer.

For SA1-35467, Production of the Main Celiac Disease Autoantigen by Transient Expression in Nicotiana benthamiana. Marín Viegas VS,Acevedo GR,Bayardo MP,Chirdo FG,Petruccelli S. Frontiers in plant science.

For 561927, Kuroki M, Matsuo Y, Kinugasa T, Matsuoka Y. Augmented expression and release of nonspecific cross-reacting antigens (NCAs), members of the CEA family, by human neutrophils during cell activation. J Leukoc Biol. 1992; 52(5):551-557.

For 16-0037-81, A library-based screening method identifies neoantigen-reactive T cells in peripheral blood prior to relapse of ovarian cancer. Martin SD,Wick DA,Nielsen JS,Little N,Holt RA,Nelson BH. Oncoimmunology.

For 16-0289-81, A mobile endocytic network connects clathrin-independent receptor endocytosis to recycling and promotes T cell activation. Compeer EB,Kraus F,Ecker M,Redpath G,Amiezer M,Rother N,Nicovich PR,Kapoor-Kaushik N,Deng Q,Samson GPB,Yang Z,Lou J,Carnell M,Vartoukian H,Gaus K,Rossy J. Nature communications.

For 563423, Van Wauwe JP, Goossens JG, Beverley PC. Human T lymphocyte activation by monoclonal antibodies; OKT3, but not UCHT1, triggers mitogenesis via an interleukin 2-dependent mechanism. J Immunol. 1984; 133(1):129-132.

For MOPC-21, Trend of telomerase activity change during human iPSC self-renewal and differentiation revealed by a quartz crystal microbalance based assay. Zhou Y et al. Scientific reports 2014 NOV.

Eukaryotic cell lines

Policy information about cell lines

Cell line source(s)	ATCC
Authentication	No authentification was performed.
Mycoplasma contamination	Cell lines used in the study were negative for Mycoplasma contamination and were regularly tested.
Commonly misidentified lines (See ICLAC register)	Not applicable.

Human research participants

Policy information about studies involving human research participants

Population characteristics

Healthy Controls N=5, Mild Patients N=10, Severe Patients (ICU) N=21.

Anagraphic values
Age, yr: Median (IQR): 66 (64-73), 69 (56-80), 67 (58-70)
Male, no. (%): 4 (80), 6 (60), 17 (81)
Coexisting disorder, no. (%):
Any : 2 (40), 10 (100), 17 (81)
Obesity: 0 (0), 2 (22), 3 (14)
Hypertension: 2 (40), 10 (100), 11 (52)
Diabetes: 0 (0), 3 (30), 7 (33)
Chronic obstructive pulmunary disease: 0 (0), 2 (20), 1 (5)
Cardiovascular disease: 0 (0), 5 (50), 3 (14)
Cronic kidney disease: 0 (0), 2 (20), 1 (5)
Active malignancies: 0 (0), 0 (0), 2 (10)

Median (IQR) interval from syntoms onset (S.O.) and Outcome
Days from S.O. to Hospitalization: -, 5 (2-6), 6 (4-7)
Days from S.O. to ICU admission:_ -, -, 7 (6-10)
Days from S.O. to dismission from ICU: -, -, 38 (23-45)
Days from S.O. to dismission from Verona Hos-pital (alive or dead): -, 21 (13-24), 43 (32-66)
Outcome, no. deaths (%): -, 1 (10), 8 (38)

Clinical features at sampling
APACHE score, Median (IQR): -, -, 23.5 (15-28.5)
SOFA score, Median (IQR): -, 2 (0.8-3.3), 6 (4-7)
pCO2 [35-40 mmHg], Median (IQR): -, 34 (31-41), 48 (41-52)
pO2 [80-100 mmHg], Median (IQR): -, 59 (55-64), 77 (69-97)
FiO2 %, Median (IQR): - 26 (21-31), 60 (50-90)
P/F ratio mmHg, Median (IQR): -, 213 (194-267), 146 (71-177)
WHO ordinal score (1-8), Median (IQR): - ,4 (3-4), 7 (7-7)

Laboratory findings at sampling, Median (IQR)
P-D-Dimer [inf. a 500µg/L]: -, 1600 (690-2501) 2250 (1319-3684)
Trombin clotting time (pt) 0,82-1,17 INR: - 1.06 (0.98-1.1) 1.14 (1.09-1.25)
P-fibrinogen [2,00 - 4,00 g/L] - 4.2 (3.3-5) 6.8 (5.4-7.8)

Microbiology analysis on BAL at sampling
Lung infection (CFU>104), no. (%) : -, - ,10 (48)
Pseudomonas lung infection (CFU>104), no. (%): -, -, 7 (33)

Recruitment

This study includes a group of 21 severe COVID-19 patients admitted to ICU, 10 mild SARS-CoV-2 patients and 5 HDs. The clinical features of the 3 groups of individuals are recapitulated in table 1. All 31 patients with COVID-19 were admitted, within the period from March 12th to April 20th 2020 to the University Hospital of Verona. At sampling, the stage of disease was categorized as mild (patients not requiring non- invasive/mechanical ventilation and/or admission to ICU, WHO ordinal score 3-4) or severe (patients requiring admission to ICU and/or non-invasive/mechanical ventilation, WHO ordinal score 6-7). All patients were hospitalized in ICU for respiratory organ failure as proved by their clinical parameters (SOFA score, pCO2, pO2, FiO2, P/F ratio).

Ethics oversight

All relevant ethical guidelines have been followed, and any necessary IRB and/or ethics committee approvals have been obtained. The study has been registered in ClinicalTrials.gov with following id NCT04438629. This study was approved by the local ethical committee (Prot. n° 17963, and n° 51095, P.I. Vincenzo Bronte). All participants (and/or initially their families) provided written in-formed consent before sampling and for the use of their clinical and biological data.

Note that full information on the approval of the study protocol must also be provided in the manuscript.

Clinical data

Policy information about clinical studies

All manuscripts should comply with the ICMJEguidelines for publication of clinical research and a completedCONSORT checklist must be included with all submissions.

Clinical trial registration

The ClinicalTrials.gov identifier of this project is protocol NCT04438629.

Study protocol

This study was approved by the local ethical committee (Prot. n° 17963, and n° 51095, P.I. Vincenzo Bronte); informed consent was obtained from all the participants to the study.

Data collection

All 31 patients with COVID-19 were admitted, within the period from March 12th to April 20th 2020 to the University Hospital of Verona. The patients were admitte to the ER than transfered to either COVID -19 department (Department of internal medicine Pulmonary/respiratory unit or ICU.
The aim of the project is to evaluate the immunological features of COVID-19 patients. Patients are recruited without any pharmacological treatments restriction. The number of samples is estimated on the basis of feasibility, that means on the maximum number of patients with COVID-19, who are expected to be able to be enrolled by the units involved. Based on the investigators' experience, gained in the onco-immunological field, considering the time and economic resources available, the investigators expect to enroll at least 80 patients.
The study aims to:
1) Determine the frequency of circulating immune cells (i.e. T cells, B cells, Neutrophils, Monocytes absolute numbers) in COVID-19 patients at Hospital Trust of Verona.
2) Determine the plasma levels of soluble factors (i.e. IL-1beta, IL-6, IL-10, TNFalfa) in COVID-19 patients at Hospital Trust of Verona.
3)Determinate the immune composition of lung and blood of COVID-19 patients that will be enrolled at Hospital Trust of Verona through single-cell sequencing analysis, capable of detecting also the viral sequences in each leukocyte populations.
4) Determine any potential links between cytokine storm, immune cells composition and clinical parameters (i.e.) in COVID-19 patients
5)Profile patients with a different stage of disease to identify potential biomarkers
6)Identify SARS-CoV-2-associated sequences in immune cells
7)To define differences in immune cell composition the immune profiling will be done before and after patients treatment used as part of clinical care in COVID-19 patients.
Once clinical care has commenced, a blood sample will be collected in three different moments: T0 = at diagnosis (which is equivalent for severe symptoms that will begin a therapeutic course, or for light symptoms within 72 hours from the COVID-19 diagnosis) ; T1= after 7 days from diagnosis (for patients admitted to the UOC the collection will be carried out at the Operative Unit while for asymptomatic patients this collection will be done at the patient's home by health workers and then the sample will be sent to the Immunology Section); T2 = after 14 days from diagnosis similar to that described for the previous collection. All the samples will be collected into vacutainer serum separator tubes.
In some cases, patients admitted to ICU also receive BAL (bronchiole-alveolar lavage) or tracheal / mini BAL aspiration. The BAL will be collected whenever it is an integral part of the patient's diagnostic path or within thirty minutes of the patient's confirmed death. In all cases, together with the BAL it will also be necessary to collect a test tube of blood, independently and in addition to the samples described above.
These samples will be analyzed for: a) a complete immune phenotype by flow cytometry; b) a panel of soluble factots (i.e. cytokines), c) a deep immunological composition by single-cell RNA sequencing, d) presence of viral sequences.
All biological materials will be stored after anonymisation at of Medicine Department of University and Hospital Trust of Verona for the duration of the study (8 months). After that, all biological materials will be transferred at Biobanca of University and Hospital Trust of Verona. All patients data will be only accessed by researchers involved in the study with a personnel account. All patients are anonymzed during immunological analysis. All data are protected by software and database of University and Hospital Trust of Verona.

Outcomes

Primary Outcome Measures:
COVID-19 associated immune disorder [Time Frame: 24 hours]
Enumeration of circulating cell subsets by flow cytometry [Cell count/μl]

COVID-19 associated inflammation [Time Frame: 48 hours]
Quantification of plasma levels of different solubles factors (GM-CSF, G-CSF, M-CSF, IFN-γ, IFN-α, IL-1, IL-2, IL-4, IL-5, IL-6, IL-9, IL-10, IL-12 (p70), IL-13, IL-15, IL-17A, IL-17F, IL-17E, IL-21, IL-22, IL-23, IL-27, IL-28A, IL-31, IL-33, IL-34, MIP-3α/CCL20, CCL2, TNF-α, TNF-β, TGFβ) [pg/ml]

Oxygenation [Time Frame: 24 hours]
Ratio of arterial oxygen tension (mmHg) to fraction of inspired oxygen (PaO2/FiO2)

Diagnostic of COVID disease composite [Time Frame: On admission of hospital]
SARS-CoV-2 infection will be tested by PCR using nasopharyngeal swab

Secondary Outcome Measures :
Changes at the cytokine pattern [Time Frame: 14 Days]
Quantification of plasma levels of different solubles factors (GM-CSF, G-CSF, M-CSF, IFN-γ, IFN-α, IL-1, IL-2, IL-4, IL-5, IL-6, IL-9, IL-10, IL-12 (p70), IL-13, IL-15, IL-17A, IL-17F, IL-17E, IL-21, IL-22, IL-23, IL-27, IL-28A, IL-31, IL-33, IL-34, MIP-3α/CCL20, CCL2, TNF-α, TNF-β, TGFβ) [pg/ml]

Changes at circulating immune cell composition [Time Frame: 14 Days]
Enumeration of circulating cell subsets by flow cytometry [Cell count/μl]

Intensive Care Unit Admission [Time Frame: Day 7-14]
Proportion of patients with Intensive Care Unit Admission requirement

Length of hospital stay [Time Frame: Day 7-14]
Days of Hospitalization

Clinical Status [Time Frame: Day 7-14]
Clinical status assessed according to the World Health Organization guideline

Mortality [Time Frame: Day 7-14]
Proportion of death patients at days

Flow Cytometry

Plots

Confirm that:

- [x] The axis labels state the marker and fluorochrome used (e.g. CD4-FITC).
- [x] The axis scales are clearly visible. Include numbers along axes only for bottom left plot of group (a 'group' is an analysis of identical markers).
- [x] All plots are contour plots with outliers or pseudocolor plots.
- [x] A numerical value for number of cells or percentage (with statistics) is provided.

Methodology

Sample preparation	For each severe patient, approximately 20ml of BAL fluid was obtained, stored at room tempera-ture and processed within 2 hours in a BSL-3 laboratory. No BAL fluid was obtained from mild patients and healthy donors. An unprocessed aliquot was used for bacterial culture. The BAL fluid was filtered 2 times through a nylon gauze and a 100-µm nylon cell strainer to remove clumps and debris. The supernatant was then washed with PBS 1x and centrifuged. RBCs were lysed with 4 mL of 0.2% NaCl solution (3 minutes, RT) and the reaction was blocked by adding 9 mL of 1.2% NaCl solution. The cells were washed with PBS 1x, re-suspended in RPMI 1640 medium supple-mented with 5% bovine serum albumin and counted. Peripheral blood (PB) from COVID-19 patients and HDs was collected in EDTA-coated tubes. 2 ml of PB was washed once with PBS 1x and the RBCs lysis was performed twice adding 15 mL of 0.2% NaCl solution (3 minutes, RT) and the reaction was blocked by adding 35 mL of 1.2% NaCl solution. The cells were washed with PBS 1x, re-suspended in RPMI 1640 medium supplemented with 5% bovine serum albumin, filtered through a 100-µm nylon cell strainer and counted.
Instrument	FACS Canto II (BD, Franklin Lakes, NJ, USA), Seegene Nimbus instrument (Seegene; Seoul, South Korea), MALDI-tof (VITEK-MS, BioMérieux; France), Glomax (Promega) plate reader, 10x Genomics Chromium Controller and the Chromium NextGEM Single Cell 3′ GEM, Library & Gel Bead kit v3.1 (Pleasanton, California, United States), Fragment Analyzer High Sensitivity NGS kit (Agilent Technologies, Santa Clara, CA, USA), Illumina NextSeq500 (Illumina, San Diego CA, USA).
Software	FlowJo software (Tree Star, Inc., Ashland, OR, USA).
Cell population abundance	The monocytes represented the 5-20 % of total PBMCs, while 80 % of total blood were represented by neutrophils. Among these cells, the LDNs represented variably 10-60 % of the cells. The NDN fraction was 5-95 % represented by neutrophils.
Gating strategy	We started from perypheral blood, isolated PBMCs by ficoll paque than analysed the cells by FACS: we recall FSC/SSC, then the FSC-A FSC-H to exclude doublets. We than recall CD14 and CD16 to define monocytes and monocytes subsets: classical monocytes CD14++ CD16 low, intermediate monocytes CD14+ CD16+, non classical monocytes CD14 low CD16++. On total monocytes we evaluated the expression of HLA-DR, ARG1 and PD-L1 by recalling SSC vs HLA-DR, SSC-ARG1 and SSC-CD274, respectively. In Fig. S2G we depicted the gating strategy. LDN neutrophils were isolated from total blood by ficoll paque gradient followed by CD66b+ magnetic sorting. The NDN neutrophils were instead isolated from total blood upon sequential ficoll paque and dextran gradient separtion, followed by CD66+ magnetic sorting.

- [x] Tick this box to confirm that a figure exemplifying the gating strategy is provided in the Supplementary Information.
